# Relationship between secondary metabolites and ecological suitability zones for *Eucommia ulmoides*

**DOI:** 10.1371/journal.pone.0317368

**Published:** 2025-01-30

**Authors:** Ting Yao, Ya Wang, Na Zhang, Beichao Wang, Zhuoting Gan

**Affiliations:** 1 Office of Scientific Research, Huangshan University, Huangshan, Anhui, China; 2 College of Life and Environment Sciences, Huangshan University, Huangshan, Anhui, China; 3 School of Chemistry and Chemical Engineering, Huangshan University, Huangshan, Anhui, China; 4 School of Tourism, Huangshan University, Huangshan, Anhui, China; Forest Survey of India, INDIA

## Abstract

The quality of Chinese medicinal materials is closely related to the types and contents of their secondary metabolites, while ecological adaptability influences the production of secondary metabolites. Therefore, identifying the relationship between ecological adaptability and secondary metabolites is important for enhancing the quality of Chinese medicinal materials. In this study, we collected 10-year-old Cortex Eucommiae (*Eucommia ulmoides*, *EU*) samples from 21 plots in eight provinces which are the primary production areas of *EU* in central China. We used the MaxEnt model to determine the ecological suitability zones for *EU* at the 21 sampling sites and classified them accordingly. The contents of six pharmacologically active secondary metabolites, including chlorogenic acid, aucubin, geniposidic acid, geniposide, pinoresinol monoglucoside, and pinoresinol diglucoside were measured in the *EU* bark. The results demonstrated significant variations in the content of the six secondary metabolites in *EU* bark among different sampling sites. Correlation analysis indicated a close relationship between the content of chlorogenic acid and aucubin in *EU* bark and the ecological suitability of their respective production areas. The total content of the six secondary metabolites also showed good consistency with the ecological suitability of the production areas. Exploratory factor analysis further revealed a strong consistency between the factor analysis comprehensive scores based on the content of major secondary metabolites and the types of suitability zones at the sample locations. The cluster analysis results demonstrated good consistency between clustering groups and ecological suitability zone groups, with higher consistency as the suitability level of the ecological zone increased. This indicated a significant impact of suitable ecological environments on the content of *EU* secondary metabolites.

## Introduction

*Eucommia ulmoides* (*EU*) is a unique and rare dioecious tree species indigenous to China [1,2]. It is classified as a relic species from the Tertiary period [3,4] and belongs to the monotypic genus *Eucommia* within the angiosperm family Eucommiaceae [5,6]. Research indicated that *EU* originated in China during the Paleogene and subsequently dispersed to various regions. With the onset of the Ice Age, it retreated from North America and Europe, eventually finding refuge in Southeast Asia, including China [7,8]. The primary production regions for *EU* are situated in central China, encompassing provinces such as Guizhou, Henan, Shaanxi, Sichuan, Hunan, Hubei, Jiangxi, and Anhui [9–11]. With the growing recognition of the uses and commercial potential of *EU*, the cultivation area has progressively expanded. Presently, the cultivated area of *EU* in China is 350,000 ha, with cultivation activities observed across 27 provinces, municipalities, and autonomous regions. Moreover, *EU* has been successfully introduced to countries including the United States, the United Kingdom, France, Hungary, Russia, Japan, and South Korea [12].

As a traditional medicine, *EU* has a history of use of more than 2000 years in China. During the Eastern Han Dynasty (25–220 AD), “Shennong’s Materia Medica Classic” listed *EU* as a superior medicinal herb [13]. The bark and leaves of *EU* are known for nourishing the liver and kidneys, strengthening tendons and bones and aiding in fetal health, as documented in the 2020 edition of the “Chinese Pharmacopoeia”. Research has shown that the pharmacological properties of *EU* are mainly attributed to its various secondary metabolites [14]. A total of 132 active ingredients, including phenols, lignans, iridoids, phenylpropanoids, and polysaccharides, have been identified in the bark, leaves, flowers, and fruits of *EU* [15,16]. Among these, lignan-type active ingredients exert antihypertensive effects by inhibiting adenosine triphosphate activity and calcium ion influx, regulating the renin–angiotensin system, dilating blood vessels, increasing coronary blood flow, and combating hypertension [13].Iridoid-type active ingredients demonstrate beneficial effects on lowering blood pressure, antioxidation, and anti-tumor activity [17]. Phenylpropanoid-type active ingredients possess antibacterial properties, tumor inhibition and free radical scavenging effects [15], while polysaccharides can enhance immunity and improve disease resistance [18].

The authenticity of medicinal materials is deeply rooted in traditional Chinese medicine theory and served as a unique standard for evaluating the quality of Chinese medicinal materials by ancient practitioners. According to traditional Chinese medicine, medicinal materials produced in specific geographical regions under certain natural conditions and ecological environments are considered to have the best quality compared with the same species of medicinal materials from other areas. They are believed to possess highly effective medicinal properties and contain the highest levels of active ingredients; thus, they are referred to as “authentic medicinal materials”. For example, *EU* produced in counties such as Lueyang in Shaanxi, Wangcang in Sichuan, Zunyi in Guizhou, Cili in Hunan, and Ruyang in Henan is designated as an “authentic medicinal material” [19]. The authenticity of *EU*, as a concept established by convention, encompasses attributes derived from history and culture, as well as various factors such as genetics, environment, and production practices.

With the expansion of *EU* cultivation areas, various ecological adaptability zones have emerged owing to differences in geographical environments, climate conditions, and other natural factors across different planting regions. Ecological suitability zoning is a regional division based on the spatial differentiation of Chinese medicinal resources and ecological environmental factors. Environmental factors within ecosystems such as geology, landforms, soil types, and climate have significant influences on the quality of Chinese medicinal materials [20]. Plants, owing to their immobility, have developed adaptive mechanisms to cope with adverse environmental conditions. For instance, adjusting the synthesis of secondary metabolites is one such suitability mechanism. Studies have shown that the cold resistance and drought resistance [21,22] of plants are sometimes attributed to the function of flavonoids or other phenolic compounds in cell walls and membranes [23].

The authenticity and quality of Chinese medicinal materials are closely related to the types and contents of their secondary metabolites and ecological adaptability can significantly influence the production of secondary metabolites. Therefore, uncovering the relationship between ecological adaptability and secondary metabolites is important. In this study, six characteristic secondary metabolites of *EU*, including chlorogenic acid, geniposidic acid, aucubin, geniposide, pinoresinol monoglucoside, and pinoresinol diglucoside, were selected [24]. The MaxEnt model was used to delineate the ecological suitability zones of the primary cultivation areas of *EU* in central China (including Anhui, Jiangxi, Henan, Hubei, Hunan, Shaanxi, Guizhou, and Sichuan) and the influence of ecological suitability zones on the six secondary metabolites of *EU* was investigated.

## Materials and methods

### Sampling sites and plant material

Bark samples from 10-year-old *EU* trees were collected in 21 counties and districts in central China with a long history of *EU* production during mid to late June 2020 (Table 1). Three healthy *EU* trees were selected at each site and the bark was harvested using a ring-barking method at a height of 1.5 m above the ground. The outer bark was removed and the inner bark was stacked to induce sweating until it turned purplish-brown. Subsequently, it was sun-dried to prepare the samples for further use.

**Table 1:** Geographic locations of *EU* sampling sites.

Plot ID	Elevation	Latitude	Longitude	Region
HNYL	109.37	28.4738	110.373	Yuanling County, Hunan Province
HNLY	67.12	28.2112	113.427	Liuyang City, Hunan Province
HNCL	74.56	29.4147	111.161	Cili County, Hunan Province
AHQM	84.04	29.7727	117.644	Qimen County, Anhui Province
ANBZ	32.44	33.8315	115.786	Bozhou City, Anhui Province
AHYX	601.24	30.8567	116.327	Yuexi County, Anhui Province
HNRY	548.59	33.894	112.467	Ruyang County, Henan Province
HNLB	977.22	34.2763	110.661	Lingbao City, Henan Province
SCWC	427.35	32.1139	106.522	Wangcang County, Sichuan Province
SCHS	2203.63	32.0648	103.026	Heishui County, Sichuan Province
GZRJ	500.25	26.0114	108.233	Rongjiang County, Guizhou Province
GZPZ	1525.47	25.7707	104.648	Panzhou City, Guizhou Province
GZZY	1002.25	27.6122	106.532	Zunyi County, Guizhou Province
HBCY	86.59	30.5045	111.178	Changyang County, Hubei Province
HBYY	202.04	32.8942	110.827	Yunyang County, Hubei Province
HBES	436.57	30.2793	109.504	Enshi City, Hubei Province
JXLS	317.29	26.698	113.945	Long City, Jiangxi Province
JXDX	62.24	28.8362	117.633	Dexing City, Jiangxi Province
SXYL	418.14	34.2595	108.06	Yangling District, Shaanxi Province
SXHL	977.96	35.6688	108.944	Huangling County, Shaanxi Province
SXLY	641.15	33.323	106.159	Lüeyang County, Shaanxi Province

### Determination of secondary metabolites in *EU* bark

#### Reagents.

Reference standards of chlorogenic acid (No.wkq20042702, ≥98.0%), aucubin (No.wkq20082603, ≥98.0%), geniposide (No.wkq00938, ≥98.0%), geniposidic acid (No.wkq20042105, ≥98.0%), pinoresinol monoglucoside (No.wkq20061112, ≥98.0%) and pinoresinol diglucoside (No.wkq20040304, ≥98%) were purchased from Sichuan Vickey Biological Technology Co., Ltd. Methanol and acetonitrile were of chromatographic grade (China National Pharmaceutical Group Corporation), while other reagents were of analytical grade. Milli-Q purified water was used for experiments.

#### Instruments and equipment.

The following instruments and equipment were used in the analysis: high-performance liquid chromatography coupled with triple quadrupole mass spectrometry (1260/6460 model, Agilent Technologies, USA), equipped with an electrospray ionization source; numerically controlled ultrasonic cleaner (JK-500DB model, Hefei Jinnike Mechanical Manufacturing Co., Ltd.); electronic balance (FA2204B model, Shanghai Youke Instrument Co., Ltd.); grinder (FS-100 model, Hebi City Minsheng Technology Development Co., Ltd.); high-speed centrifuge (TG16 model, Hunan Kecheng Instrument Equipment Co., Ltd.).

#### Sample preparation.

The *EU* bark samples were dried at 45°C, ground, sieved through a 60-mesh sieve and 0.20 g of powder was weighed and placed in a 25-mL conical flask. Then, 4.00 mL of 80% methanol was added, the flask was sealed with filter paper and ultrasonicated at room temperature for 60 min (power: 500 W, frequency: 40 kHz, with a 10-min rest period after every 10 min of sonication). After cooling to room temperature, any mass loss was compensated with 80% methanol, followed by filtration. The filtrate was centrifuged at 16,000 rpm for 10 min and the supernatant was transferred to a 1.5-mL vial for analysis.

#### Reference standard solutions.

Reference standards of chlorogenic acid, aucubin, geniposidic acid, pinoresinol monoglucoside, geniposide, and pinoresinol diglucoside were individually weighed at 1.125 mg, 1.011 mg, 0.997 mg, 1.128 mg, 1.098 mg and 1.210 mg, respectively. Each standard was dissolved in methanol and made up to a final volume of 10 mL in a volumetric flask. The solutions were filtered through a 0.2-μm membrane filter and kept for further use.

#### Chromatographic conditions.

The chromatographic conditions were as follows: chromatographic column: Waters Acquity HSS T3 (2.1 mm ×  100 mm, 1.8 μm); flow rate: 0.3 mL/min; column temperature: 30°C; injection volume: 1 μl. The mobile phase consisted of 0.1% formic acid solution (A) and acetonitrile (B). The gradient elution conditions were as follows: 0–1 min, 5% A; 1–10 min, 5%–11% A; 10–23 min, 11%–15% A; 23–45 min, 15%–26% B; 45–51 min, 26%–41% A; 51–57 min, 41%–60% A; 57–57.5 min, 60%–100% A.

#### Mass spectrometric conditions.

The electrospray ionization source was used in positive ionization mode and the mass scan range was m/z 50–1500. Capillary voltage was set at 3 kV; cone voltage was set at 40 V; ion source temperature was 100°C; desolvation gas temperature was 500°C; desolvation gas flow rate was 800 L/h; low-energy collision voltage was set at 6 eV and high-energy collision voltage was set at 20–30 eV.

#### Methodology investigation.

##### Standard curve.

A mixed reference solution was prepared by mixing 1 mL of each standard solution. A stock solution based on similar proportions to the mixed reference solution was then diluted in concentration gradients of 5-fold, 10-fold, 20-fold, and 50-fold. The peak area of each standard compound was plotted on the vertical axis (Y), while the sample concentration was plotted on the horizontal axis (X), resulting in the standard curves for the six standard compounds (Table 2).

**Table 2:** Linear relationships, linear range, precision, stability, repeatability and recovery of the analysis method.

Compound	Linear equation	Correlation coefficient *r*	Linear range (μg/mL)	Precision (RSD,%)	Stability (RSD,%)	Repeatability (RSD,%)	Recovery (%)	Recovery (RSD,%)
Chlorogenic acid	y = 60787x – 31994	0.9992	0.38–112.50	4.74	15.23	2.00	111.85	2.84
Geniposidic acid	y = 4414.1x + 13973	0.9881	0.34–101.10	2.66	6.62	1.17	119.37	3.86
Geniposidic	y = 14975x - 678.51	0.9991	0.34–99.70	1.82	11.74	3.10	111.16	2.37
Aucubin	y = 3061.2x + 4454.9	0.9998	0.38–112.80	1.05	7.29	2.97	118.22	2.96
Pinoresinol monoglucoside	y = 2737.7x + 13986	0.9826	0.37–109.80	2.48	20.32	2.73	86.18	3.71
Pinoresinol diglucoside	y = 6095.3x–12841	0.9913	0.40–121.00	1.59	15.90	2.43	105.90	1.67

##### Precision test.

The HNYL *EU* bark sample was prepared to make the test solution. Six consecutive measurements were taken, and the peak areas were recorded. The relative standard deviation (RSD) was calculated. The RSD of the peak areas for the six active compounds ranged from 1.05% to 4.74%, indicating good instrument precision (Table 2).

##### Stability test.

The HNCL *EU* bark sample was prepared to make the test solution. Samples were injected and tested at the following time points: 0 h, 2 h, 4 h, 6 h, 8 h, 10 h, 12 h, 14 h, 16 h, 18 h, 20 h, 22 h, and 24 h. The peak areas were recorded, and the RSD was calculated. The RSD of the peak areas for the six main active compounds ranged from 6.62% to 20.32%, indicating that the method had good stability (Table 2).

##### Repeatability test.

Six samples of the SCWC *EU* bark, each weighing 0.2 g, were accurately weighed, and test solutions were prepared. The samples were injected and analyzed, and the peak areas were recorded. The RSD of the content for each component was calculated. The RSD of the peak areas for the six active compounds ranged from 1.17% to 3.10%, indicating good repeatability of the method (Table 2).

##### Spike recovery test.

Six samples of SCWC *EU* bark, each weighing 0.06 g, were taken to prepare the test solutions. Then, 40 µ L of chlorogenic acid, 240 µ L of geniposide acid, 240 µ L of geniposide, 120 µ L of aucubin, 120 µ L of pinoresinol monoglucoside, and 300 µ L of pinoresinol diglucoside were mixed. The samples were injected and analyzed. The recovery rate and RSD were calculated. The results showed that the recovery rates for the six active compounds ranged from 86.18% to 119.37%, and the RSD of the peak areas ranged from 1.67% to 3.86%, indicating good accuracy of the method (Table 2).

#### Identification of secondary metabolites.

Data were collected using UPLC-Q-TOF-MS/MS technology and the obtained mass spectra were analyzed using a MassLynx 4.1 workstation. Each peak ion flow diagram in the positive ion mode was cross-checked and fragment ions were derived based on the compound fragmentation pattern. A chemical composition database was established by consulting relevant literature. The molecular formula of compounds was inferred based on the accurate relative molecular mass obtained from the main chromatographic peaks. The molecular weight and molecular structure of compounds were identified and attributed to each chromatographic peak.

### *EU* ecological suitability zoning

#### Prediction of ecologically suitable areas for *EU* based on the MaxEnt model.

MaxEnt was proposed by E.T. Jaynes [25] in the 1950s, with its core idea being to select the probability distribution with maximum entropy under the constraints of known facts. When using MaxEnt to predict species distribution, the constraints are derived from the environmental variable characteristics of the species’ actual geographic distribution points, and the model seeks the possible distribution with maximum entropy under these constraints [26]. The probability distribution of species occurrence at maximum entropy is the closest to the actual distribution of the species. This model estimates species distribution probabilities in different environments by considering known constraints and with minimal prior assumptions. It is one of the most widely used tools in species distribution and ecological niche modeling, with good predictive performance.

In this study, climate, soil, and topography were used as constraint conditions, with the assumptions that the probability of species presence was equal in suitable habitats and background environments, and that species could freely disperse within their suitable areas without geographic or ecological barriers. The MaxEnt model was used to predict the probability distribution of *Eucommia*. The delineation and assessment of the ecologically suitable areas for *Eucommia* are significant for the conservation of *Eucommia* resources, introduction and cultivation, refined management, and sustainable use. In the optimal suitable areas, cultivation areas can be expanded, while large-scale artificial planting in unsuitable areas is not advisable. Additionally, efforts should be made to enhance the diversity protection of *Eucommia* germplasm resources in unsuitable regions.

#### Data sources and processing.

The *EU* specimen distribution data within the study area were obtained from the Chinese Virtual Herbarium (http://www.cvh.ac.cn/) and the Global Biodiversity Information Facility (http://www.gbif.org/, https://doi.org/10.15468/dl.tnnk6g). After excluding specimens with incomplete or duplicate location information, 296 remaining sample data points were obtained. Geographic coordinates were retrieved using the Tencent Map Coordinate Picker (https://lbs.qq.com/getPoint/) (S1 Table). Nineteen climate variables were obtained from the WorldClim 2.0 database (https://www.worldclim.org/) with a spatial resolution of 2.5′. Thirty-five soil variables were obtained from the Harmonized World Soil Database v2.0 [27] (https://www.fao.org/) with a spatial resolution of 30″. Three terrain variables were provided by Geospatial Data Cloud site, Computer Network Information Center, Chinese Academy of Science (http://www.gscloud.cn). After clipping with ArcGIS software, the study area shapefile was obtained, which was then used to extract climate, soil, and terrain variables within the study area. In modeling species distribution, multicollinearity among environmental variables can result in overfitting of the species distribution model, thereby reducing the accuracy of the predictions [28,29]. Therefore, Spearman’s rank correlation analysis was performed to assess the correlations among climate variables and soil variables (S1 and S2 Figs). Variables with correlation coefficients less than 0.8 were retained. For variables with correlation coefficients > 0.8, the variable with the smaller average correlation coefficient with other variables was retained. Finally, 33 variables were selected for modeling (S2 Table).

#### *EU* ecological suitability zoning using SDMtoolbox.

SDMtoolbox is an ArcGIS plugin that can be used in conjunction with MaxEnt [30]. The *EU* sample data (including sample ID, longitude, and latitude) were saved in CSV format, while climate variables, soil variables and terrain variables were converted to ASCII format and imported into SDMtoolbox. The training set was randomly selected from 75% of the sample data, with the remaining 25% as the test set [31]. The modeling process was repeated 10 times (replicates), with the remaining parameters set to default values. The environmental factor contributions were assessed using the Jackknife method and model accuracy was evaluated using Receiver Operating Characteristic (ROC) curves. The area under the ROC curve (AUC) was calculated as an evaluation index of model performance. After 10 repetitions, the average AUC values for the training and test data sets were 0.812 and 0.749, respectively (S3 Fig), indicating a good fit of the model to the distribution data of *EU* [32].

Ecological suitability zoning was conducted using the Reclassify tool in ArcGIS 10.2 spatial analysis tools. On the basis of the ecological suitability probability (P–value) obtained from the MaxEnt model and considering the actual distribution of *EU*, the potential geographic distribution of *EU* was divided into five levels: unsuitable (p <  0.01), low suitability (0.01 ≤ p <  0.25), moderate suitability (0.25 ≤ p <  0.5), high suitability (0.5 ≤  p <  0.75) and optimal suitability (0.75 ≤  p < 1).

## Results

### Geographic distribution characteristics of the six secondary metabolites

*EU* contains a variety of secondary metabolites, including phenols, sesquiterpenes, lignans, steroids, terpenes and flavonoids [13,16]. Among them, chlorogenic acid in the phenol class, aucubin and geniposidic acid in the sesquiterpene class, along with their derivatives geniposide and lignans such as pinoresinol monoglucoside and pinoresinol diglucoside are the main constituents of *EU* bark and serve as important chemical markers and characteristic components [15].

**Fig 1:**
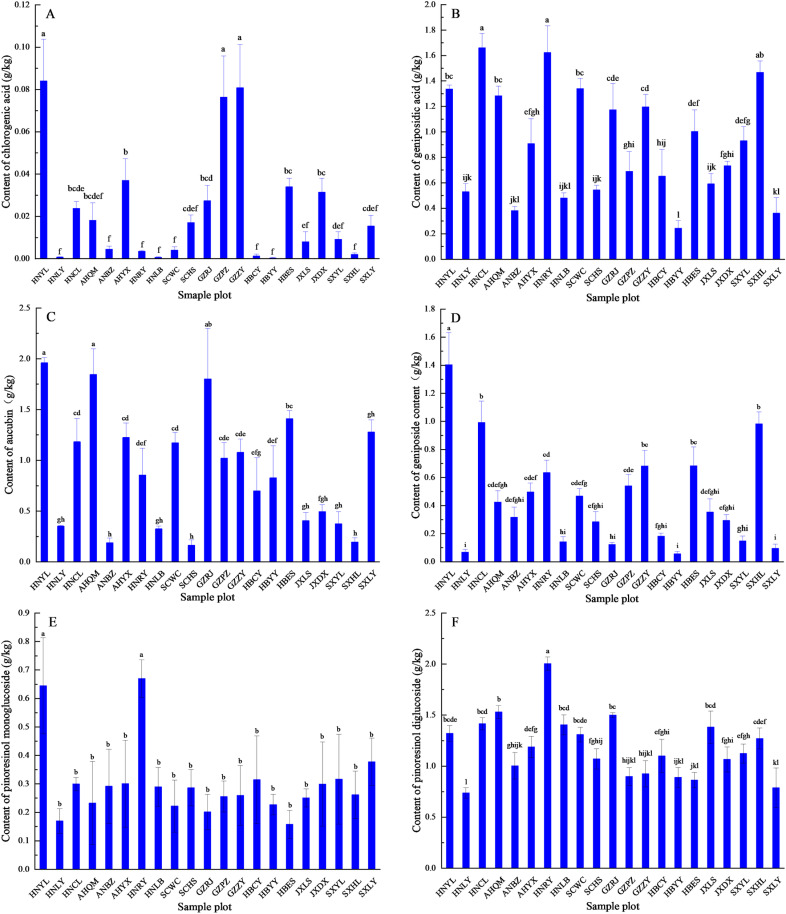
Distribution of chlorogenic acid (A), geniposidic acid (B), aucubin (C), geniposide (D), pinoresinol monoglucoside (E); F: pinoresinol diglucoside (F) in *EU* bark samples. The full names of the horizontal coordinate sample sites can be found in Table 1. Different lowercase letters on the bar chart indicated highly significant differences (*p* <  0.01).

In this study, the content of chlorogenic acid in *EU* bark ranged from 0.003 g/kg to 0.084 g/kg, with an average of 0.0228 g/kg. There were significant differences in chlorogenic acid content among different sampling points (Fig 1A). Variance analysis showed that the chlorogenic acid content in the bark samples from Yuanling, Zunyi, and Panzhou was significantly higher at 0.084 g/kg, 0.081 g/kg and 0.076 g/kg, respectively, compared with other regions (*p* <  0.01). Yuexi also exhibited significantly higher chlorogenic acid content than the remaining regions (*p* <  0.01). Qimen, Heishui, Luoyang, Yangling, Longshi, Bozhou, Wangcang, Ruyang, Huangling, Changyang, Liuyang, Lingbao, and Yunyang showed no significant difference (*p* <  0.01).

The content of geniposidic acid in *EU* bark ranged from 0.24 g/kg to 1.66 g/kg, with an average of 0.91 g/kg, showing significant differences among sampling sites (Fig 1B). In areas such as Cili, Ruyang, Huangling, Wangcang, Yuanling, Qimen, Zunyi, Rongjiang, and Enshi, the content of geniposidic acid was > 1.00 g/kg. There were significant differences (*p* <  0.01) in geniposidic acid content between Cili, Ruyang and Yuanling, as well as between Qimen and Enshi. In contrast, in areas such as Yangling, Yuexi, Dexing, Panzhou, Changyang, Longshi, Heishui, Liuyang, Lingbao, Bozhou, Luoyang, Lueyang and Yunyang, the geniposidic acid content was < 1.00 g/kg.

The content of aucubin in *EU* bark ranged from 0.16 g/kg to 1.95 g/kg, with an average of 0.90 g/kg, showing significant differences among different locations (Fig 1C). In areas such as Yuanling, Qimen, Rongjiang, Enshi, Lueyang, Yuexi, Cili, Wangcang, and Zunyi, the content of aucubin was > 1.00 g/kg. There were significant differences (*p* <  0.01) in aucubin content between Yuanling and Enshi, Rongjiang, Yuexi, Cili, Wangcang, and Zunyi. In contrast, in areas such as Panzhou, Ruyang, Yunyang, Dexing, Longshi, Yangling, Lueyang, Luyang, Lingbao, Huangling, Bozhou, and Heishui, the content of aucubin was < 1.00 g/kg.

The maximum content of geniposide in *EU* bark at each site ranged from 0.24 g/kg to 1.66 g/kg, with an average of 0.91 g/kg. There were significant differences in geniposide content among the samples (Fig 1D). The site in Yuanyang had the highest geniposide content of 1.40 g/kg, which was significantly higher than other sites (p <  0.01).

The content of pinoresinol monoglucoside in *EU* bark samples ranged from 0.16 g/kg to 0.67 g/kg, with an average of 0.30 g/kg. There were significant differences in pinoresinol monoglucoside content between samples from Ruyang and Cili, compared with other samples (*p* <  0.01) (Fig 1E).

The maximum content of pinoresinol diglucoside in *EU* bark samples was 2.01 g/kg, while the lowest was 0.74 g/kg in Liuyang, with an average of 1.18 g/kg. There was not much fluctuation in pinoresinol diglucoside content among different sites (Fig 1F). Overall, apart from Ruyang, where the content of pinoresinol diglucoside in *EU* bark reached 2.01 g/kg, the content in Qimen, Rongjiang, Cili, Lingbao, Longshi, Yuanyang, Wangcang, Huangling, Yuexi, Yangling, Changyang, Heishui, Dexing, and Bozhou was above 1.00 g/kg, while it was below 1.00 g/kg in Zunyi, Panzhou, Yunyang, Enshi, Lueyang, and Liuyang.

Overall, the distribution of the six main compounds in *EU* bark varied in the study area, with significant differences in coefficient of variation. chlorogenic acid showed the greatest variation, followed by aucubin, geniposide, geniposidic acid and pinoresinol monoglucoside, while the variation range of pinoresinol diglucoside was the smallest.

### Distribution of ecological habitat zones for EU sampling sites

The geographic coordinates of the sampling points were imported into the distribution map of ecological habitat zones for *EU* in central China, to create an ecological habitat zone distribution map of the 21 sampling points. ArcGIS 10.2 was used to extract the ecological adaptability probability (P-value) for each *EU* sampling point and identify the ecological habitat zone level for each sampling point (Table 3). The ecological habitat zone levels for each sampling point were as follows: Heishui, Huangling, Dexing, Bozhou, and Yangling were classified as low suitability zones; Lingbao, Longshi, Changyang, Wangcang, Yunyang, and Ruyang were classified as moderate suitability zones; Liuyang, Yuexi, Lueyang, Cili, and Rongjiang were classified as high suitability zones; Enshi, Zunyi, Qimen, Yuanyang, and Panzhou were classified as the optional suitability zones.

**Table 3:** Geographical information and ecological suitability of *EU* sampling plots.

Plot ID	P-value (MaxEnt Model)	Suitable Zone	Suitable Type Number
SCHS	0.03	Low suitability zone	L1
SXHL	0.10		L2
JXDX	0.10		L3
AHBZ	0.17		L4
SXYL	0.19		L5
HNLB	0.30	Moderate suitability zone	M1
JXLS	0.32		M2
HBCY	0.37		M3
SCWC	0.38		M4
HBYY	0.40		M5
HNRY	0.41		M6
HNLY	0.53	High suitability zone	H1
AHYX	0.56		H2
SXLY	0.64		H3
HNCL	0.65		H4
GZRJ	0.68		H5
HBES	0.75	Optimal suitability zone	O1
GZZY	0.75		O2
AHQM	0.79		O3
HNYL	0.77		O4
GZPZ	0.80		O5

### Relationship between ecological suitability of *EU* origin and secondary metabolites in *EU* bark

Ecological suitability reflects the degree to which organisms adapt to their growth and ecological environment and quantifies the interaction between organisms and their habitat. Adjusting the synthesis of secondary metabolites is an adaptive mechanism of plants to their environment. Plants can produce different components and levels of secondary metabolites in different environments, leading to differences in their intrinsic qualities [33]. Pearson correlation analysis between the ecological suitability probability (P-value) of *EU* origin and the content of six major secondary metabolites in *EU* bark, including chlorogenic acid, geniposidic acid, aucubin, geniposide, pinoresinol monoglucoside, and pinoresinol diglucoside, was conducted to explore the differences in the effects of the environmental suitability at the *EU* origin on the content of these secondary metabolites.

**Fig 2:**
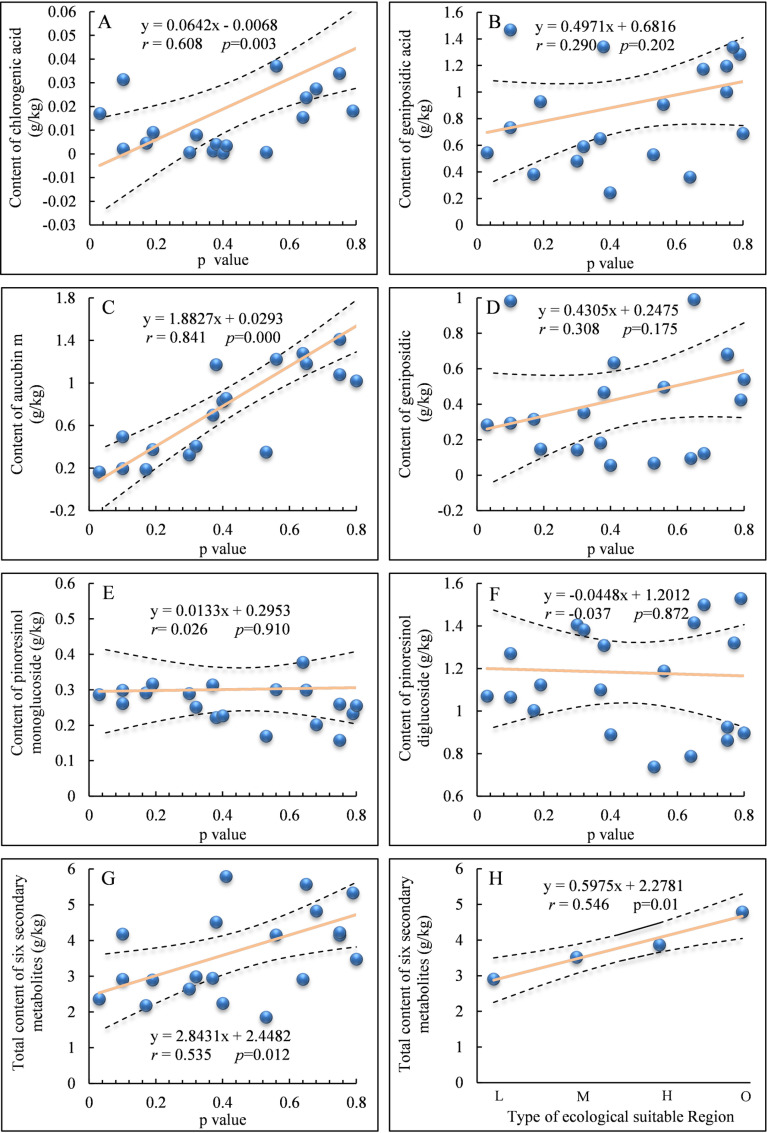
Relationship between P-value of the ecological suitability of *EU* producing regions and the content of chlorogenic acid (A), geniposide acid (B), aucubin (C), geniposidic (D), pinoresinol monoglucoside (E) and pinoresinol diglucoside (F) in EU bark at the 21 sampling sites. Relationship between the total content of six secondary metabolites in bark and P-value of the ecological suitability of EU producing regions at the 21 sampling sites (G). The distribution of the average total content of six secondary metabolites in four ecological suitability zones (H).

Fig 2A–F shows the linear fit of the content of chlorogenic acid, geniposidic acid, aucubin, geniposide, pinoresinol monoglucoside, and pinoresinol diglucoside in EU bark with the corresponding P-value at 21 origin sites. The content of aucubin was highly positively correlated with the ecological suitability P-value of the origin sites, with a Pearson correlation coefficient (*r*) of 0.841 (*p* <  0.01). The content of chlorogenic acid was also significantly positively correlated with the ecological suitability P-value of the origin sites, with an *r* of 0.608 (*p* <  0.01). However, the content of geniposidic acid and geniposide showed weak and non-significant correlations with the ecological suitability P-value of the origin sites, with an *r* of 0.290 and 0.308, respectively Similarly, there was no significant correlation between the content of pinoresinol monoglucoside and pinoresinol diglucoside and the ecological suitability P-value of the origin sites. Fig 2G depicts the *r* between the total content of all six secondary metabolites and the ecological suitability P-value of the origin sites, which is 0.535 (*p* <  0.05), indicating a significant moderate correlation. Fig 2H presents the distribution of the mean values of the total content of the six secondary metabolites in the four ecological suitability zones (Low, Moderate, High, and Optimal). With the increase in ecological suitability level, the mean total content of the six major secondary metabolites in *EU* bark showed a linear increase. Spearman rank correlation analysis showed an *r* of 0.983 (*p* <  0.01) between the ecological suitability level at the origin sites and the mean total content of the six major secondary metabolites, indicating a highly significant moderate correlation.

Overall, the content of aucubin and chlorogenic acid in *EU* bark was closely related to the ecological suitability at its origin sites. There was also a strong correlation between the total content of the main secondary metabolites in *EU* bark and the ecological suitability types of the origin sites.

### Exploratory factor analysis

Exploratory Factor Analysis (EFA) is a type of factor analysis primarily used to explore and understand the potential relationships among variables [34]. In the initial stages of data analysis, the number of underlying factors in the data is often unclear. EFA reduces the dimensionality of the data and identifies the latent factors that are present [35]. In this study, EFA was used to filter out potential factors related to six secondary metabolites in *Eucommia* bark, thereby enhancing our understanding of the classification of secondary metabolites and their responsiveness to ecologically suitable areas. A matrix of 21 × 6 order was constructed using the content of six secondary metabolites in the bark samples from 21 *EU* producing regions. The data were then imported into SPSS 25.0 software, where the original data were standardized. The EFA was conducted by selecting—Dimension Reduction—Factor Analysis, choosing coefficients and significance levels for description and selecting the maximum variance method for rotation. The Kaiser–Meyer–Olkin (KMO) measure of sampling adequacy and Bartlett’s test of sphericity were used to assess the suitability of the analysis. The KMO index ranges from 0 to 1, where 0.50 is considered suitable for factor analysis and Bartlett’s test of sphericity should be significant (*p* <  0.01) for factor analysis to be appropriate. As shown in Table 4, the KMO value was 0.53 and the Barlett’s test value was *p* <  0.01, indicating suitability for factor analysis.

**Table 4:** Kaiser-Meyer-Olkin measure of sampling adequacy and Bartlett’s test of sphericity.

Item		Value
Kaiser-Meyer-Olkin Measure of Sampling Adequacy		0.530
Bartlett’s Test of Sphericity	Approx. Chi Square	54.423
	df	15
	Sig.	0.000

Using the criterion of eigenvalues greater than 1 for extraction, the eigenvalue of Principal Component 1 (PC1) was 2.898, with a variance contribution rate of 48.303%. The eigenvalue of the second principal component (PC2) was 1.404, contributing to 23.394% of the variance. The cumulative contribution rates of PC1 and PC2 was 71.697%. After rotation, the eigenvalue of the first principal component was 2.243, contributing to 37.392% of the variance and the eigenvalue of the second principal component was 2.058, contributing to 34.305% of the variance. The cumulative variance contribution rate remained at 71.697% (Table 5). Typically, a cumulative variance contribution rate greater than 70% is considered satisfactory [36], indicating that PC1 and PC2 contained most of the information regarding the composition of *EU* bark compounds. Therefore, these two principal components were used for comprehensive evaluation of the compound composition of *EU* bark in different regions.

**Table 5:** Total variance explained.

Component	Initial Eigenvalues			Extraction Sums of Squared Loadings			Rotation Sums of Squared Loading		
	Eigenvalue	% of Variance	Cumulative/%	Eigenvalue	% of Variance	Cumulative/%	Eigenvalue	% of Variance	Cumulative/%
1	2.898	48.303	48.303	2.898	48.303	48.303	2.243	37.392	37.392
2	1.404	23.394	71.697	1.404	23.394	71.697	2.058	34.305	71.697
3	0.768	12.797	84.494						
4	0.605	10.082	94.576						
5	0.217	3.613	98.189						
6	0.109	1.811	100.000						

**Fig 3:**
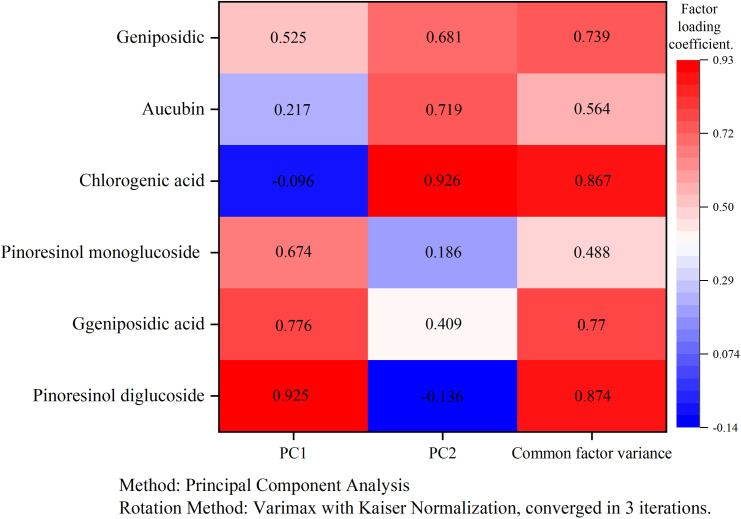
Heat map of factor loading rotation matrix.

From the rotated factor loading matrix heatmap (Fig 3), it can be observed that pinoresinol diglucoside, geniposidic acid, and pinoresinol monoglucoside had higher loadings on PC1. These three compounds have proven to have significant antihypertensive effects. Therefore, the first factor F1 mainly explains the antihypertensive components of *EU*. In contrast, chlorogenic acid, aucubin and geniposide exhibited higher loadings on PC2. These three compounds possess anti-tumor properties. Hence, the second factor F2 primarily explains the anti-tumor components of *EU*.

From Table 6, the factor score coefficient matrix (estimated by regression) was obtained, yielding the factor score functions:

**Table 6:** Component score coefficient matrix.

Component	Factor	
	1	2
Chlorogenic acid	-0.217	0.528
Geniposidic acid	0.318	0.084
Aucubin	-0.021	0.357
Geniposide	0.142	0.280
Pinoresinol monoglucoside	0.307	-0.020
Pinoresinol diglucoside	0.493	-0.244


$${\text{F}}_{\text{1}}=-0.217\times {\text{Z}}_{\text{CGA}}+0.318\times {\text{Z}}_{\text{GA}}-0.021\times {\text{Z}}_{\text{AU}}+0.142\times {\text{Z}}_{\text{GP}}+0.307\times {\text{Z}}_{\text{PM}}+0.493\times {\text{Z}}_{\text{PD}}$$

$${\text{F}}_{\text{2}}=0.528\times {\text{Z}}_{\text{CGA}}+0.084\times {\text{Z}}_{\text{GA}}+0.357\times {\text{Z}}_{\text{AU}}+0.280\times {\text{Z}}_{\text{GP}}-0.020\times {\text{Z}}_{\text{PM}}-0.244\times {\text{Z}}_{\text{PD}}$$

Subsequently, the comprehensive score values of *EU* samples at each sampling point were calculated as follows:


$$\text{F}=\left(0.37392/0.71697\right)\times {\text{F}}_{1}+\left(0.34305/0.71697\right)\times {\text{F}}_{\text{2}}$$

**Fig 4:**
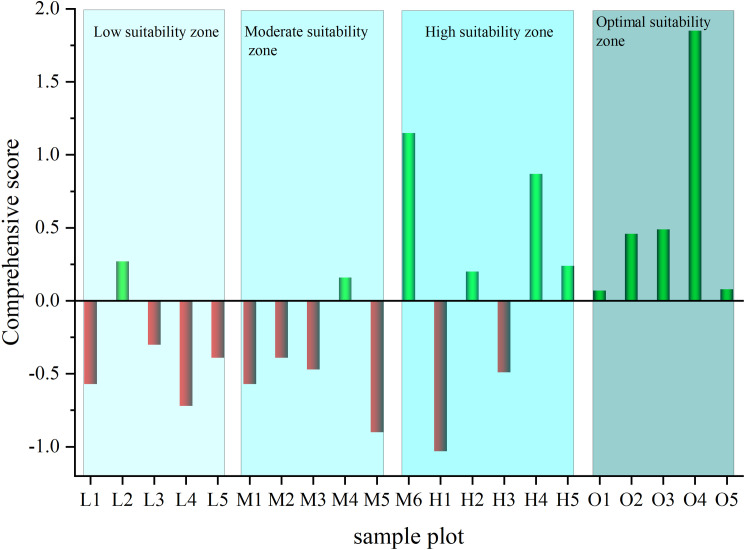
Comprehensive score of secondary metabolites from *EU* bark in 21 plots. The full names of the horizontal coordinate sample plots can be found in Table 3.

Fig 4 shows the distribution of the comprehensive score values of the main secondary metabolites of *EU* bark at the 21 sampling sites. It can be observed from the figure that, except for sites L2 and M4, the comprehensive score values of *EU* bark secondary metabolites in both the low suitability zone and the moderate suitability zone were less than 0. Furthermore, except for sites H1 and H3, the comprehensive score values of *EU* bark secondary metabolites in both the high suitability zone and the optimal suitability zone were greater than 0. Overall, the factor analysis comprehensive scores based on the content of main secondary metabolites in *EU* bark exhibited good consistency with the types of suitable ecological zones of the *Eucommia* samples, indicating that the ecological environment conducive to *Eucommia* growth enhanced the main compound composition in *EU* bark, thereby improving the quality of *Eucommia*.

### Cluster analysis

Cluster analysis is a statistical method for grouping random objects into categories and it is used when there is no priori hypothesis. Here it was used as the most appropriate sorting method to uncover differences in genetic messages. Cluster analysis includes a collection of methods that generally use multivariate and quantitative measurements to group objects or events together according to their similarity [37]. There are two types of hierarchical clustering: Relational clustering (R clustering) and Qualitative clustering (Q clustering). R clustering is variable clustering [38] and it groups variables which have similar characteristics and separates different ones. Q clustering is sample clustering. It groups samples which have similar characteristics and separates different samples [39]. We chose representative variables for analysis to reduce the number and dimension of variables.

**Fig 5:**
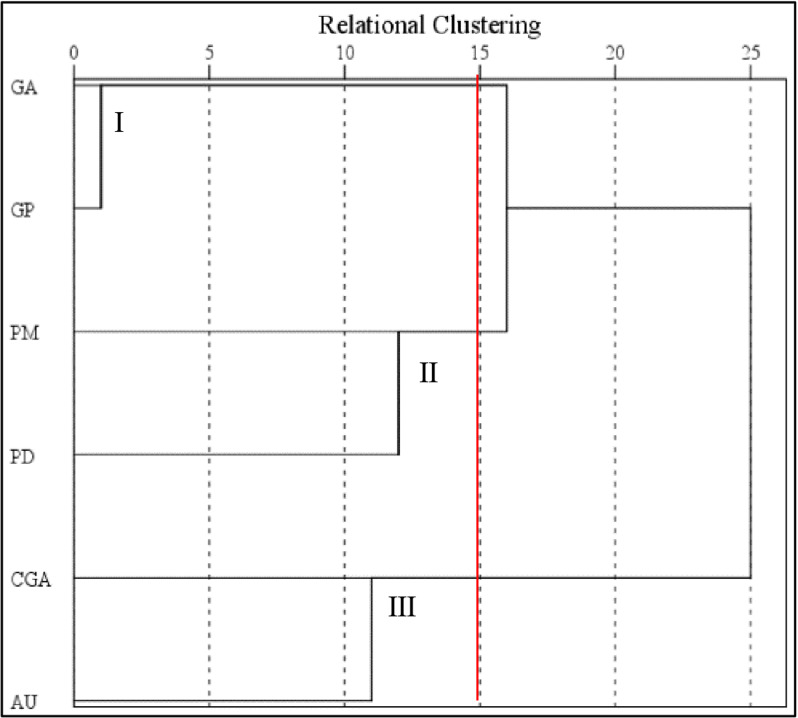
Dendrogram of the six compounds in *EU* bark grouped by R clustering. The vertical coordinates GA, GP, PM, PD, CGA and AU represent the abbreviations for geniposidic acid, geniposide, pinoresinol monoglucoside, pinoresinol diglucoside, chlorogenic acid and aucubin, respectively. I, II, and III are the cluster labels, respectively. The red dotted line indicates the cluster separation line.

This study initially performed R clustering on six active ingredients of *EU* bark in 21 sample plots, followed by Q clustering to group plots. For the R clustering of the six active ingredient variables, we selected Furthest Neighbor as the cluster method, Pearson Correlation as the measure-interval and Z score as the transform value (by variable) using SPSS. The dendrogram in Fig 5 represents the clustering results of the six active ingredient variables in *EU* bark. According to the dendrogram-based clustering, the six active ingredient indicators were divided into three clusters. Cluster I included geniposidic acid and geniposide, Cluster II included pinoresinol monoglucoside and pinoresinol diglucoside and Cluster III included chlorogenic acid and aucubin. From these three clusters of chemical composition indicators, one indicator was selected from each cluster for Q clustering. In this study, the chosen indicators were geniposidic acid, pinoresinol monoglucoside, and aucubin from Clusters I, II, and III, respectively.

For the Q clustering of 21 sample plots, we first standardized the content of six secondary metabolites in geniposidic acid, pinoresionol monoglucoside and aucubin, then selected the Furthest Neighbor as the cluster method, Pearson Correlation as the measure-interval and Z score as the transform value (by variable) using SPSS. The clustering dendrogram is depicted in Fig 7. The 21 sample plots of *EU* were divided into four clusters: I, II, III, and IV.

**Fig 6:**
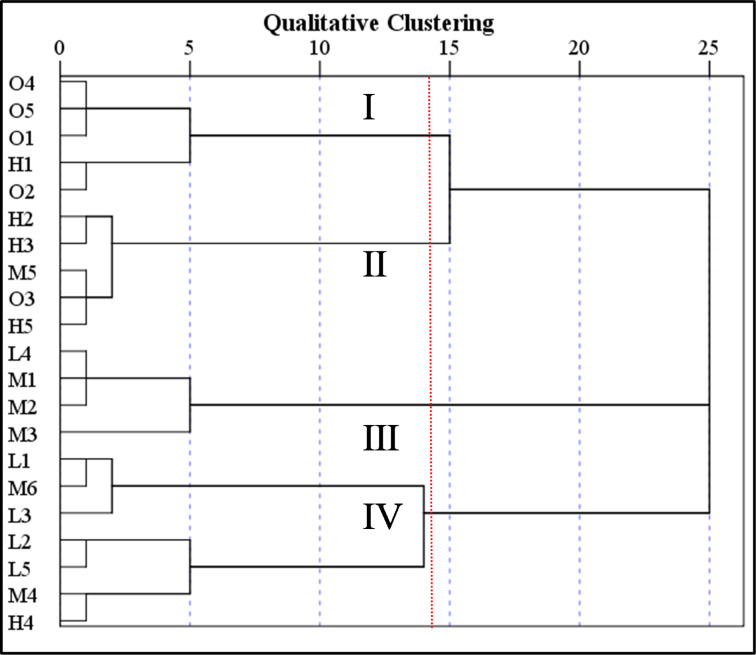
Dendrogram of the cases among 21 sample plots of *EU* grouped by Q clustering. I, II, III, and IV are classified as cluster numbers. The red dotted line indicates the cluster separation line.

According to the dendrogram of the 21 sample plots by Q clustering (Fig 6) and the ecological suitability of *EU* sampling plots (Table 3), we compared the Q-cluster partition and ecological adaptability zones of 21 *EU* sample plots (Table 7). Table 7 shows that Cluster I included *EU* plots O1, O2, O4, and O5 in the optimal suitability zone and plot H1 in the high suitability zone. The matching accuracy between Cluster I *EU* plots and those in the optimal suitability zone reached 80%. Cluster II included *EU* plot O3 in the optimal suitability zone, plots H2, H3, and H5 in the high suitability zone and plot M5 in the moderate suitability zone. The matching accuracy between Cluster II *EU* plots and those in the high suitability zone was 60%. Cluster III included *EU* plots M1, M2, M3, and M6 in the moderate habitat zone and plot L4 in the low suitability zone. The matching accuracy between Cluster III *EU* plots and those in the moderate suitability zone was 75%. Cluster IV included *EU* plots M4 and M6 in the suitability habitat zone and plots L1, L2, L3 and L5 in the low habitat zone. The matching accuracy between Cluster IV *EU* plots and those in the low suitability zone was 57%. Overall, there was good consistency between clustering groups and ecological suitability zone groups.

**Table 7:** Comparison of cluster types and ecological zone types.

Cluster type	Sample plot						Suitable Type	Matching accuracy^*^
I	O1	O2	O4	O5	H1		Optimal suitability	80%
II	O3	H2	H3	H5	M5		High suitability	60%
III	M1	M2	M3	L4			Moderate suitability	75%
IV	M4	M6	L1	L22	L3	L4	Low suitability	57%

* Matching accuracy is the Ecological Zone types/the cluster type of sample plots.

## Discussion

### *EU* species migration and secondary metabolites

Since the Eocene, *Eucommia’s* northern boundary fluctuated with global temperatures. It decreased from 55°N to 47°N in the Eocene–Oligocene cooling, peaked at 58°N in the late Oligocene-middle Miocene warming, then retreated to 52°N in the late Miocene–Pliocene cooling. Post-Quaternary glacial activities saw it retreat to 35°N.The preservation of *Eucommia* through geological periods of north–south migration demonstrates its remarkable environmental adaptability. Plant migration is typically accomplished through changes in intergenerational distribution zones and migration entails crossing land barriers to enter new environments [40,41]. If plant populations or entire species fail to adapt and keep pace with environmental changes, they may decline or become extinct [42–44]. Confronted with various adversities, plants must possess mechanisms to resist unfavorable environments while maintaining their health, gradually evolving specific mechanisms to adapt to adversity, namely secondary metabolites. The ecological role of plant secondary metabolites is primarily to enhance adaptability. Plants have evolved to produce large amounts of secondary metabolites only under adverse conditions, which is considered the optimal strategy for plant competition [33]. Because plant individuals cannot move freely, brief environmental stresses can also rapidly induce significant changes in secondary metabolism [45]. Compounds such as flavonoids or other phenolic compounds in secondary metabolites are sometimes attributed to the cold and drought resistance of plants [23]. Salvia miltiorrhiza Bunge [46], chlorogenic acid, catechins, polyphenols [47], flavonoids [48] and other secondary metabolites are related to soil moisture deficiency. The composition of short chains in plants is significantly influenced by temperature, with almost all plant species exhibiting increased short chains with rising temperatures [49]. The composition of phenols [50] and terpenes [49]is also positively correlated with temperature.

**Fig 7:**
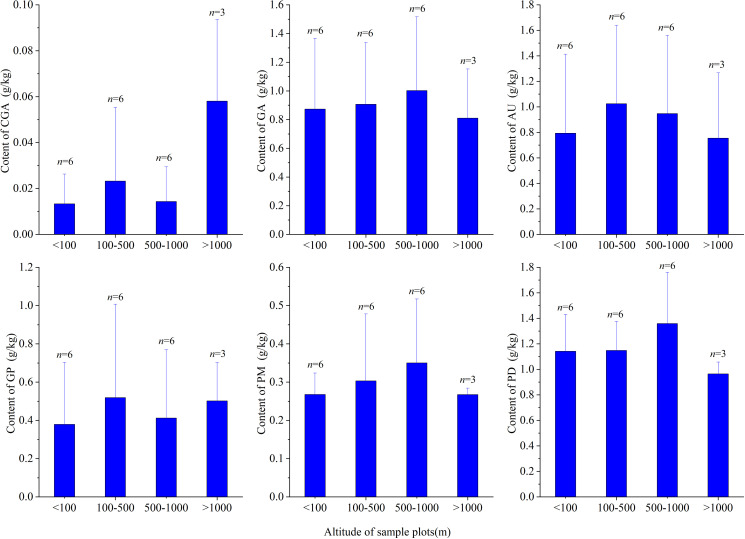
The average content distribution of chlorogenic acid (A), geniposidic acid (B), aucubin (C), geniposidic (D), pinoresinol monoglucoside (E), pinoresinol diglucoside (F) in the *EU* sample plots at four altitude segments: <100 m, 100 m–500 m, 500 m–1000 m, and >1000 m. The *n* above the bar represents the number of *EU* sample plots located in the four altitude ranges.

In this study, the 21 sampling sites were classified into four altitude categories: 0–100 m (n = 6), 100–500 m (n = 6), 500–1000 m (n = 6) and > 1000 m (n = 3). The distribution of the average content of six secondary metabolites in *EU* bark samples at each altitude interval is shown in Fig 7. In high-altitude (>1000 m) sampling sites, the content of chlorogenic acid significantly increased, while the contents of aucubin and pinoresinol monoglucoside were relatively low. The differences in the content of the six secondary metabolites in *EU* bark samples were small at altitudes below 1000 m, with slightly higher contents of the six secondary metabolites at moderate altitudes (100–1000 m) than at low altitudes (<100 m). It can be inferred that the increase in chlorogenic acid content in *EU* bark samples at high altitudes (>1000 m) is due to the low-temperature environment and is related to *Eucommia’s* mechanism of adjusting the content of secondary metabolites to adapt to the environment.

Through the analysis of the migration history of *Eucommia* species and the elevation distribution of secondary metabolites in the sampled areas, the global migration and preservation of *Eucommia* during geological periods demonstrated its strong environmental adaptability. Secondary metabolites are key mechanisms by which plants respond to adversity. Elevation and temperature significantly influenced the regulation of secondary metabolites in *Eucommia*, providing theoretical support for the selection of cultivation areas and the enhancement of quality. This has important implications for further understanding the mechanisms by which plants cope with environmental changes.

### Relationship between geo-authentic herbs and ecologically suitable areas

The concepts of “authenticity” and “geo-authenticity” are commonly used in traditional Chinese medicine. “Geo-authentic” medicinal herbs refer to those grown in specific regions and with unique production methods. It is generally believed that geo-authentic medicinal herbs have higher quality compared with non-geo-authentic ones [51]. High levels of effective components are the main identification feature of geo-authentic herbs [45].

Ecological suitability refers to the size of the living space provided by a specific ecological environment for a particular biological community and the suitability for positive succession. Species exhibit preferences for habitats and the more suitable the habitat, the more likely they are to be distributed in that area, thus forming ecological suitability zones for species in geographical space. The zoning of ecological adaptability is mainly based on the geographical spatial division of species ecological adaptability factors such as climate, soil, vegetation type, and historical record data.

For Chinese medicinal materials, habitats not only produce changes in the appearance of medicinal materials, but also directly affect secondary metabolites (many of which are bioactive components), resulting in differences in medicinal properties [52]. Both the geo-authenticity and ecological suitability of Chinese medicinal materials are influenced to varying degrees by the habitat. Therefore, there is a correlation between the geo-authenticity and ecological suitability of Chinese medicinal materials.

However, the geo-authenticity of Chinese medicinal materials proposed by traditional Chinese medicine is mainly defined and distinguished based on the efficacy of the medicinal materials, while ecological suitability is also related to factors such as survival rate and incidence rate of diseases in medicinal materials [53]. Chinese medicinal materials grown in environmentally suitable areas may not necessarily exhibit optimal medicinal effects. Owing to the strong resistance of plants, some secondary metabolites of medicinal materials grown in stressful environments may increase rather than decrease, thus producing high-quality medicinal materials. Although suitable environmental conditions are important factors for producing high-quality Chinese medicinal materials, medicinal materials grown in ecologically suitable areas should not be equated with geo-authentic Chinese medicinal materials [54]. In this study, the content of chlorogenic acid, geniposidic acid, aucubin, and geniposide in *EU* bark was positively correlated with the ecological suitability zone, while the content of geniposide and geniposidic acid showed no significant correlation with ecological suitability zones.

The secondary metabolites of plants play important roles in regulating growth, metabolism, and immunity, as well as in chemical communication between plants, resisting pests and adverse stress, and promoting soil microbial activity and nutrient cycling. The formation, accumulation, and variation of secondary metabolites are influenced by their habitats. For medicinal plants, the secondary metabolites within the plant are crucial in determining the yield and quality of traditional Chinese medicine materials. Among the 616 types of medicinal materials and decoction pieces included in the 2020 edition of the “Pharmacopoeia of the People’s Republic of China,” the majority, 535 species (86.85%) [55], were of plant origin. Therefore, clarifying the relationship between the ecological environment and the secondary metabolites in plant-derived traditional Chinese medicine materials can enhance the yield and quality of these materials by selecting and constructing suitable ecological environments. This allows for the targeted cultivation of medicinal plants with high levels of desired secondary metabolites and aids in the optimization of medicinal material production locations and the breeding of high-activity varieties.

Although the MaxEnt model has been widely used for ecological adaptability zoning, the selection of *Eucommia* specimens, data quality, and the relevance of environmental variables used as model constraints significantly impacted the zoning results. The assumptions of the MaxEnt model, such as plant distributions being in a state of equilibrium and free dispersal, may not align with the actual conditions of *Eucommia*. Additionally, the content of secondary metabolites in *Eucommia* is subject to dynamic changes, leading to variations in measurement results owing to factors like tree age and sampling time. Therefore, the relationship between *Eucommia*’s secondary metabolites and ecological adaptability zones requires further and more in-depth research.

## Conclusion

In the main production areas of *EU* in central China, spanning eight provinces, bark samples from 10-year-old trees were collected from 21 sites and analyzed for the content of six secondary metabolites with significant pharmacological effects: chlorogenic acid, geniposidic acid, aucubin, geniposide, pinoresinol monoglucoside, and pinoresinol diglucoside. The MaxEnt model was used to delineate the ecological suitability zones for *EU* in the study area. Correlation analysis, factor analysis and cluster analysis were conducted to explore the relationship between the content of secondary metabolites in *EU* bark and the ecological suitability zones of their respective production areas, as well as to investigate the influence of *EU* habitats on the content of secondary metabolites in its bark. The following conclusions were drawn:

1. There were significant variations in the distribution of *EU* content among the eight provinces in central China, with chlorogenic acid exhibiting the greatest variability, followed by geniposide, aucubin, geniposidic acid, pinoresinol monoglucoside, and pinoresinol diglucoside, which showed the smallest amplitude of change.
2. Correlation analysis revealed a close relationship between the content of aucubin and chlorogenic acid in *EU* bark and the ecological adaptability of their respective production areas. Additionally, there was good consistency between the total content of the six main secondary metabolites and the ecological adaptability types of the production areas.
3. Factor analysis indicated that the habitats conducive to the growth of suitable *EU* are advantageous for enhancing the composition of the main compounds in *EU* bark, resulting in higher quality *EU*.
4. The cluster analysis results demonstrated good consistency between the clustering groups and the ecological suitability zone groups. Furthermore, as the level of ecological suitability zones increased, the consistency became higher, indicating that suitable ecological environments have a significant impact on the content of secondary metabolites in *EU*.

## Supporting information

S1 Fig Correlation coefficient table of 19 climate variables. (TIF)

S2 Fig Correlation coefficient of 34 soil variables. (TIF)

S3 Fig Results of the receiver operating characteristics curve. (TIF)

S1 Table *Eucommia ulmoides* specimen information. (PDF)

S2 Table Variables used in the model prediction. (PDF)
