# Supplementary figures and images for: Relationship between secondary metabolites and ecological suitability zones for Eucommia ulmoides
Source: PLoS One. 2025 Jan 30;20(1):e0317368. doi: 10.1371/journal.pone.0317368 (PMC11781706; doi:10.1371/journal.pone.0317368)

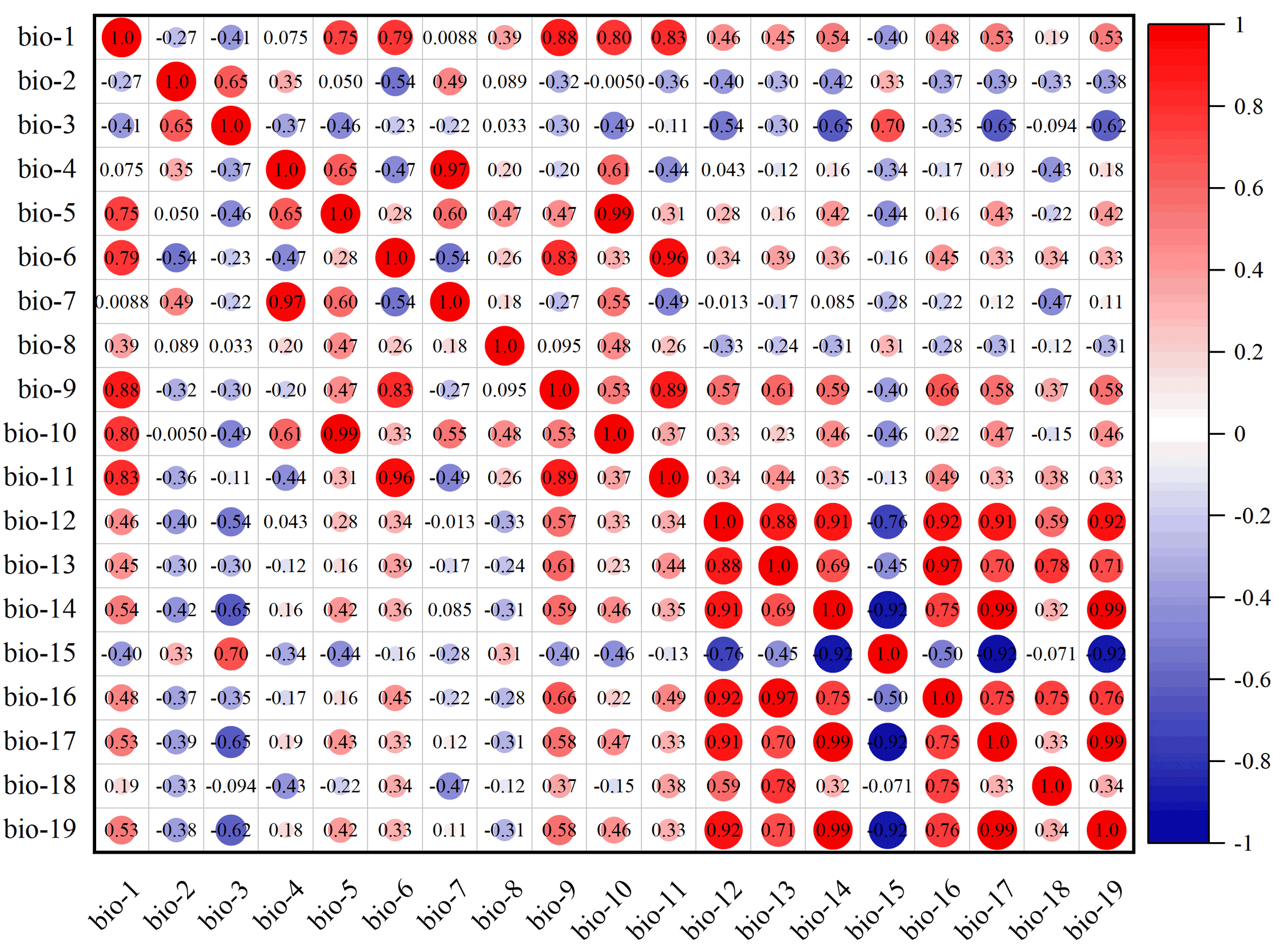

Supplement: S1 Fig — (TIF) [file pone.0317368.s001.tif]

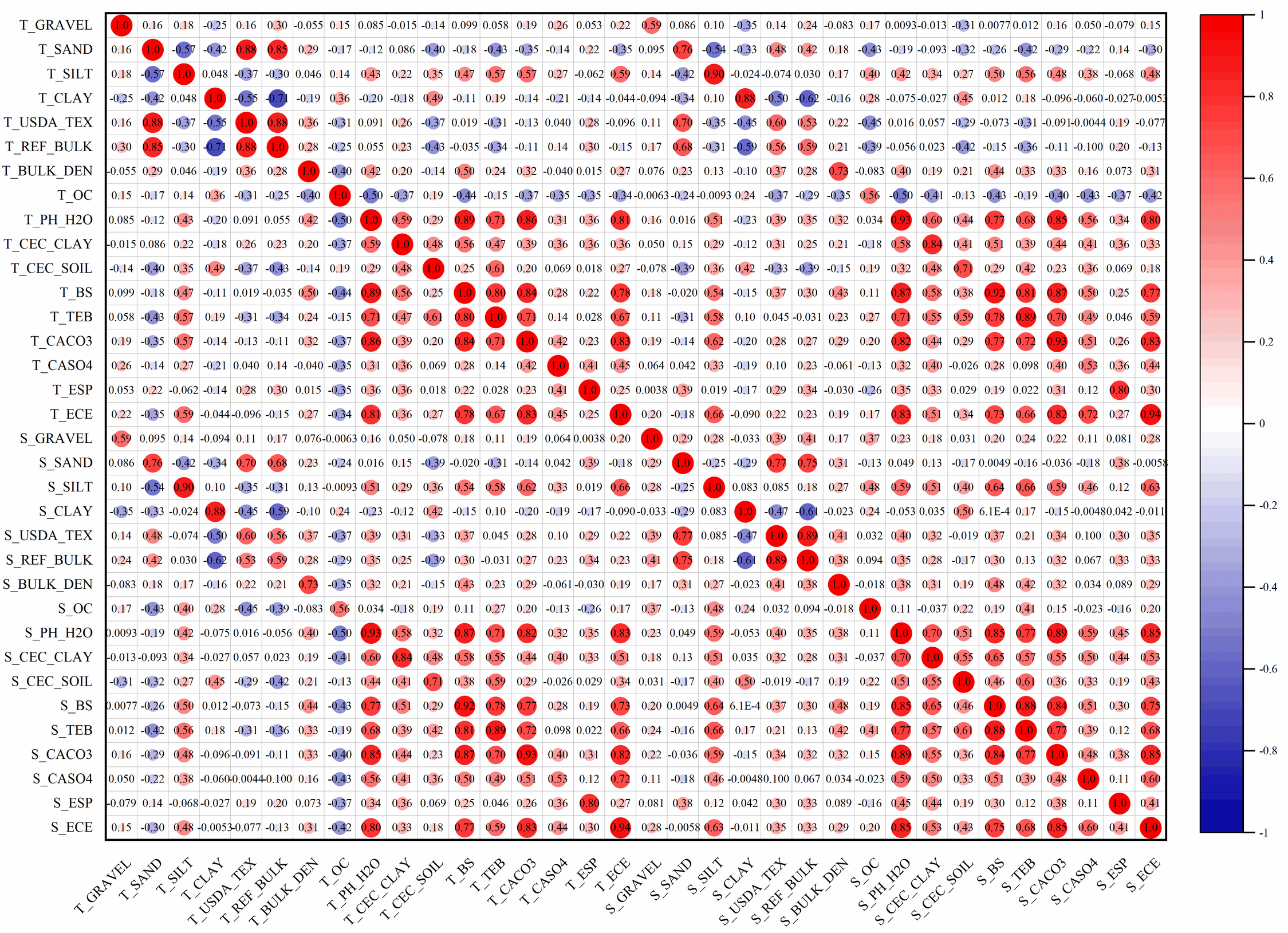

Supplement: S2 Fig — (TIF) [file pone.0317368.s002.tif]

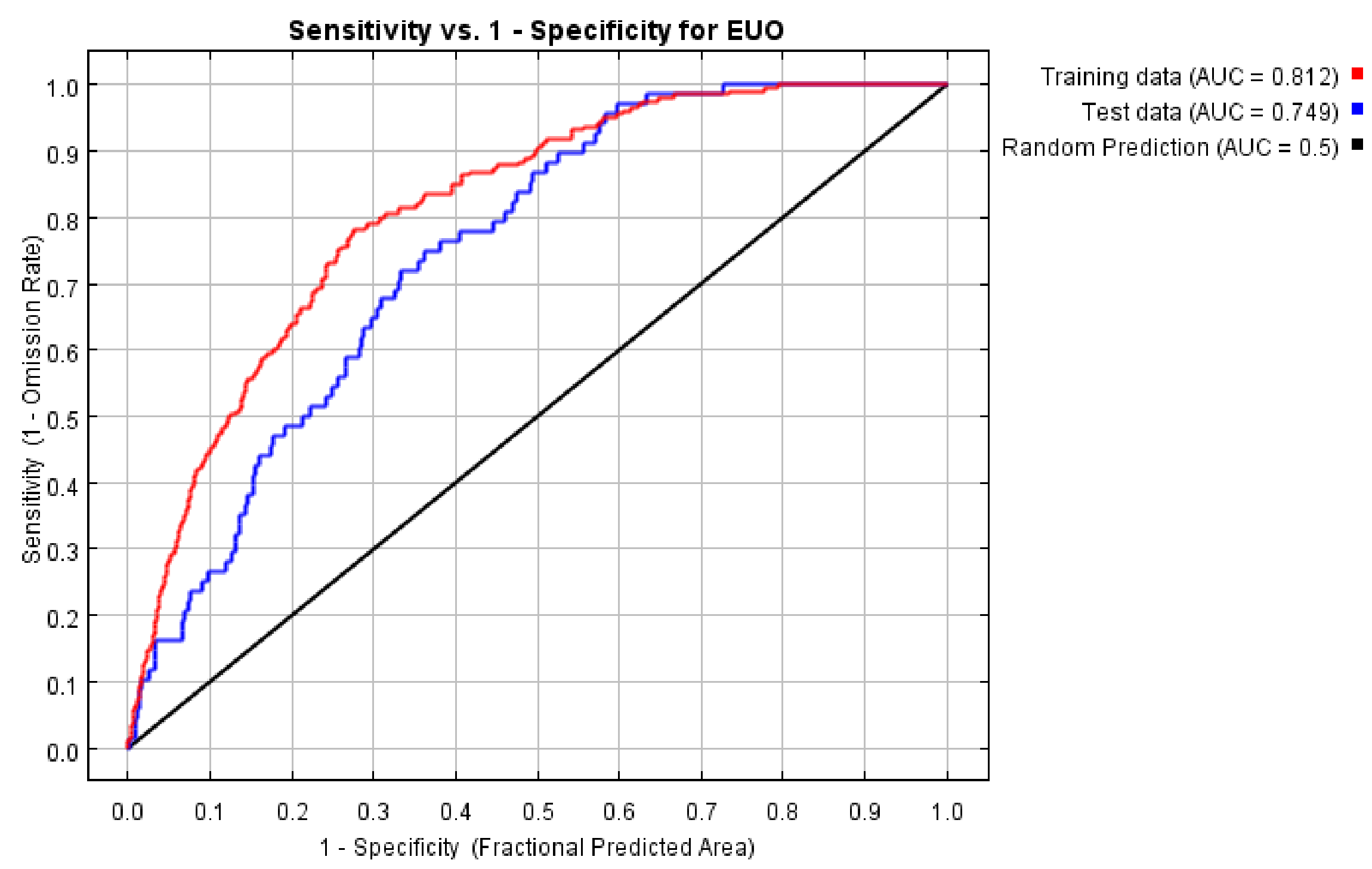

Supplement: S3 Fig — (TIF) [file pone.0317368.s003.tif]
