# Supplementary material for: Relationship between secondary metabolites and ecological suitability zones for Eucommia ulmoides
Source: PLoS One. 2025 Jan 30;20(1):e0317368. doi: 10.1371/journal.pone.0317368 (PMC11781706; doi:10.1371/journal.pone.0317368)
Supplement: S1 Table — (PDF) [file pone.0317368.s004.pdf]

S1 Table Eucommia ulmoides specimen information

location	dd altitude	species	dd longitude	dd latitude
Guichi District, Anhui Province	200	N 1	117.5757	30.36221
Jinzhai County, Anhui Province	378	N 2	115.6955	31.27178
Jing County, Anhui Province	121	N 3	118.5536	30.5743
Qimen County, Anhui Province	500	N 4	117.5729	30.03252
Shitai County, Anhui Province	700	N 5	117.2606	30.24888
Wuhu City, Anhui Province	47	N 6	118.3	31.026
Yi County, Anhui Province	500	N 7	117.9512	30.04819
Xuanchengjing County, Anhui Province	130	N 8	118.41	30.55595
Anshun City, Guizhou Province	1285.1	N 9	106.0727	26.13031
Anshun City, Guizhou Province	1200	N 10	106.3072	25.4415
Bayang Town, Guizhou Province	1216.7	N 11	106.1736	25.93241
Bijie City, Guizhou Province	1650.2	N 12	105.4018	27.44506
Chishui City, Guizhou Province	600	N 13	106.1426	28.61962
Dejiang County, Guizhou Province	547	N 14	108.215	28.28436
Guanling County, Guizhou Province	1540	N 15	105.5353	25.77667
Guanling County, Guizhou Province	1185	N 16	105.5834	25.95761
Guanling County, Guizhou Province	1191.7	N 17	105.5843	25.95656
Guanling County, Guizhou Province	646.3	N 18	105.7202	25.78864
Guiding County, Guizhou Province	1824.1	N 19	104.5567	25.90575
Guiding County, Guizhou Province	910	N 20	107.3331	26.34151
Guiding County, Guizhou Province	1204	N 21	107.3331	26.34151
Guiding County, Guizhou Province	1086	N 22	107.3331	26.34151
Guiyang City, Guizhou Province	1119	N 23	106.6703	26.40982
Guiyang City, Guizhou Province	1180	N 24	106.6731	26.46405
Guiyang City, Guizhou Province	1300	N 25	106.728	26.63068
Guiyang City, Guizhou Province	1244	N 26	106.6922	26.59832
Hezhang County, Guizhou Province	1766.9	N 27	104.7244	27.23272
Hezhang County, Guizhou Province	1880	N 28	103.8104	26.87933
Jiangkou County, Guizhou Province	851.5	N 29	108.8643	27.62144
Jiangkou County, Guizhou Province	2099	N 30	108.775	27.84389
Jiangkou County, Guizhou Province	500	N 31	108.8393	27.69965
Jingping County, Guizhou Province	400.3	N 32	109.1758	26.51791
Jingping County, Guizhou Province	494.2	N 33	109.1057	26.41753
Kaili City, Guizhou Province	970	N 34	107.47	25.44778
Kaili City, Guizhou Province	1240	N 35	106.8639	26.79729
Libo County, Guizhou Province	425	N 36	108.0729	25.39025
Libo County, Guizhou Province	568	N 37	107.949	25.2393
Liupanshui City, Guizhou Province	1190.6	N 38	105.4004	26.32039
Longli County, Guizhou Province	1334.7	N 39	106.7034	26.81936
Luodian County, Guizhou Province	999.9	N 40	107.0336	25.53376

Majiang County, Guizhou Province	1102.3	N 41	106.9679	26.46565
Puding County, Guizhou Province	1560.2	N 42	105.9257	26.47933
Rongjiang County, Guizhou Province	502	N 43	108.2202	25.9414
Rongjiang County, Guizhou Province	740	N 44	108.1978	26.18778
Sansui County, Guizhou Province	750	N 45	108.6111	26.99083
Sansui County, Guizhou Province	650	N 46	108.5772	26.89917
Sansui County, Guizhou Province	575	N 47	108.7125	26.9575
Sansui County, Guizhou Province	608	N 48	108.7303	26.86556
Shiqian County, Guizhou Province	510	N 49	108.1497	27.32347
Shiqian County, Guizhou Province	1200	N 50	108.0633	27.33353
Shiqian County, Guizhou Province	1250	N 51	108.1509	27.34838
Shiqian County, Guizhou Province	1255	N 52	108.219	27.38647
Songtao County, Guizhou Province	1120	N 53	108.7186	28.39583
Suiyang County, Guizhou Province	1205	N 54	107.1888	27.94741
Suiyang County, Guizhou Province	860	N 55	107.1042	28.25631
Alsophila Natural Reserve,Guizhou Province	862.9	N 56	105.9622	28.54667
Tongzi County, Guizhou Province	650	N 57	106.8381	28.47898
Tongzi County, Guizhou Province	1170	N 58	106.8652	28.23418
Xishui County, Guizhou Province	1298	N 59	106.3039	28.25833
Xingyi City, Guizhou Province	1462	N 60	105.0012	25.27167
Xiuwen County, Guizhou Province	1200	N 61	106.6711	26.88444
Xiuwen County, Guizhou Province	1256	N 62	106.694	26.94081
Yinjiang Province, Guizhou Province	837	N 63	108.7276	28.1217
Zhenfeng County, Guizhou Province	1252	N 64	105.4989	25.43039
Zhijin County, Guizhou Province	1326.4	N 65	106.1374	26.70669
Zunyi City, Guizhou Province	1404.2	N 66	107.5771	29.1484
Zunyi County, Guizhou Province	828.8	N 67	106.7304	27.49028
Huixian County, Henan Province	608	N 68	113.5636	35.555
Jiyuan City, Henan Province	1200	N 69	112.2775	35.1983
Lushi County, Henan Province	800	N 70	114.5479	27.59893
Lushi County, Henan Province	1060	N 71	111.0389	33.59819
Pingdingshan City, Henan Province	708	N 72	112.2991	33.71021
Xixia County, Henan Province	964	N 73	111.6491	33.67475
Xinxian County, Henan Province	223	N 74	114.7822	31.66008
Xinxiang City, Henan Province	155	N 75	113.8431	35.4725
Xinxiang City, Henan Province	73	N 76	113.8803	35.2925
Xinxiang City, Henan Province	181	N 77	113.9231	35.42694
Xinxiang City, Henan Province	75	N 78	113.8833	35.29889
Xinxiang City, Henan Province	70	N 79	113.9097	35.33639
Xinxiang City, Henan Province	83	N 80	113.9197	35.32667
Xinxiang City, Henan Province	71	N 81	113.9056	35.36278
Xinxiang City, Henan Province	70	N 82	113.9097	35.33639
Xinxiang City, Henan Province	1128	N 83	113.6381	35.61167

Xinxiang City, Henan Province	155	N 84	113.8431	35.4725
Xinxiang City, Henan Province	73	N 85	113.8803	35.29528
Xinxiang City, Henan Province	75	N 86	113.8833	35.29889
Xinxiang City, Henan Province	70	N 87	113.9097	35.33639
Xinxiang City, Henan Province	73	N 88	113.8844	35.34056
Yuzhou City, Henan Province	110	N 89	113.4966	34.16918
Zhenping County, Henan Province	187	N 90	112.2739	33.12996
Zhengzhou City, Henan Province	104	N 91	113.5357	34.81731
Zunyi City, Henan Province	200	N 92	114.0851	31.80365
Zunyi City, Henan Province	650	N 93	114.0851	31.80365
Badong County, Hubei Province	1690	N 94	109.6546	30.01779
Badong County, Hubei Province	1740	N 95	109.6546	30.01779
Shennongjia Forest Area in Hubei Province	1032	N 96	110.5814	31.43028
Baokang County, Hubei Province	600	N 97	111.1856	31.55134
Fang County, Hubei Province	700	N 98	110.7268	32.04017
Lichuan City, Hubei Province	1100	N 99	108.6698	30.47761
Shennongjia District, Hubei Province	950	N 100	110.6617	31.74699
Shennongjia District, Hubei Province	900	N 101	110.2387	31.62237
Shennongjia District, Hubei Province	750	N 102	110.3005	31.46254
Shennongjia District, Hubei Province	1350	N 103	110.3853	31.43734
Shennongjia District, Hubei Province	800	N 104	110.8916	31.59301
Wuchang City, Hubei Province	28	N 105	114.3604	30.53326
Wuchang City, Hubei Province	43	N 106	114.3086	30.54299
Wuhan City, Hubei Province	25	N 107	114.3555	30.4744
Xiantao City, Hubei Province	900-1000	N 108	113.2792	30.20585
Xingshan County, Hubei Province	263	N 109	110.6926	31.30022
Yichang City, Hubei Province	1250	N 110	110.9542	31.04269
Yidu City, Hubei Province	213	N 111	111.1918	30.2352
Yidu City, Hubei Province	213	N 112	111.1918	30.2352
Andong County, Hunan Province	270	N 113	111.6094	26.44159
Baojing County, Hunan Province	478.09	N 114	109.4038	28.66229
Baojing County, Hunan Province	304	N 115	115.6342	29.66678
Baojing County, Hunan Province	420	N 116	109.5833	28.74305
Baojing County, Hunan Province	350	N 117	109.3797	28.60088
Baojing County, Hunan Province	700	N 118	109.3797	28.60088
Chenzhou City, Hunan Province	240	N 119	113.0586	25.82725
Cili County, Hunan Province	726	N 120	110.5316	29.35819
Cili County, Hunan Province	270	N 121	111.1636	29.59165
Dong'an County, Hunan Province	300	N 122	111.3724	26.2779
Guzhang County, Hunan Province	658	N 123	110.1575	29.1167
Hengnan County, Hunan Province	450	N 124	112.7432	27.23149
Jishou City, Hunan Province	670	N 125	109.755	28.3091
Jishou City, Hunan Province	197	N 126	109.7256	28.28771

Liuyang City, Hunan Province	710-750	N 127	114.1682	28.43476
Luxi County, Hunan Province	189.5	N 128	110.0704	28.39133
Luxi County, Hunan Province	151	N 129	109.9629	28.26669
Pingjiang County, Hunan Province	165	N 130	113.8989	28.61449
Qidong County, Hunan Province	300	N 131	111.9688	26.66946
Shaoyangsi, Hunan Province	670	N 132	110.8196	26.28521
Shimen County, Hunan Province	400	N 133	110.9071	29.99614
Suining County, Hunan Province	436	N 134	110.0936	26.40798
Xiangxi Prefecture, Hunan Province	995	N 135	110.086	28.65763
Xiangxi Prefecture, Hunan Province	562	N 136	110.3148	28.92123
Xinning County, Hunan Province	450	N 137	110.7656	26.34875
Xupu County, Hunan Province	500	N 138	110.5884	28.09498
Yanling County, Hunan Province	1400	N 139	114.0444	26.49174
Yanling County, Hunan Province	740	N 140	114.0376	26.54352
Yongshun County, Hunan Province	682	N 141	110.2828	28.90283
Yongshun County, Hunan Province	431	N 142	110.2411	28.75611
Yongshun County, Hunan Province	1112	N 143	110.1555	28.96832
Yongshun County, Hunan Province	550	N 144	110.1398	28.95119
Yongshun County, Hunan Province	378	N 145	109.9067	29.22448
Yongshun County, Hunan Province	500	N 146	109.8982	29.20096
yongzhou city, Hunan Province	366	N 147	111.8198	24.9003
Yuanling County, Hunan Province	530	N 148	110.4365	28.75944
Yuanling County, Hunan Province	600	N 149	110.4365	28.75944
Changsha City, Hunan Province	50	N 150	112.9945	28.13379
Changsha City, Hunan Province	1400	N 151	112.9977	28.13315
Anfu County, Jiangxi Province	153	N 152	114.2536	27.39722
Anyuan County, Jiangxi Province	360	N 153	115.3528	25.27389
Chongyi County, Jiangxi Province	364	N 154	114.1625	25.78222
Dexing City, Jiangxi Province	29	N 155	117.4956	29.03472
Duchang County, Jiangxi Province	48	N 156	116.1903	29.25682
Fenyi County, Jiangxi Province	371	N 157	114.8763	28.0492
Fenyi County, Jiangxi Province	150	N 158	114.7169	27.76917
Ganzhou City, Jiangxi Province	558	N 159	116.4333	25.58333
Ganzhou City, Jiangxi Province	269	N 160	114.6	25.31667
Ji'an City, Jiangxi Province	981	N 161	114.0658	26.21222
Jinggangshan City, Jiangxi Province	249	N 162	114.2723	26.60292
Jiujiang City, Jiangxi Province	535	N 163	115.9755	29.54497
Jiujiang City, Jiangxi Province	531	N 164	115.2389	29.54
Jiujiang City, Jiangxi Province	41	N 165	116.0451	29.44823
Jiujiang County, Jiangxi Province	50	N 166	115.9042	29.59598
Jiujiang County, Jiangxi Province	40	N 167	115.6342	29.66678
Lianhua County, Jiangxi Province	606	N 168	114.037	27.35259
Lushan City, Jiangxi Province	1200	N 169	115.9867	29.54691

Pengze County, Jiangxi Province	300	N 170	116.5697	29.89956
Quannan County, Jiangxi Province	249	N 171	114.5301	24.74235
Ruichang City, Jiangxi Province	400	N 172	115.4392	29.69666
Ruichang City, Jiangxi Province	400	N 173	115.4756	29.71244
Ruichang City, Jiangxi Province	300	N 174	115.4392	29.69666
Ruichang City, Jiangxi Province	400	N 175	115.4392	29.69666
Shangyou County, Jiangxi Province	887	N 176	114.0531	25.91778
Shangyou County, Jiangxi Province	530	N 177	114.1881	26.015
Tonggu County, Jiangxi Province	337	N 178	114.5604	28.65458
Tonggu County, Jiangxi Province	556	N 179	114.6148	28.61116
Tonggu County, Jiangxi Province	500	N 180	114.6727	28.63039
Wuning County, Jiangxi Province	332	N 181	114.6246	29.3393
Wuning County, Jiangxi Province	500	N 182	114.9222	28.96316
Wuning County, Jiangxi Province	1550	N 183	114.9883	29.08865
Wuning County, Jiangxi Province	1250	N 184	114.9553	28.87863
Wuning County, Jiangxi Province	1300	N 185	114.9883	29.08865
Xinxi County, Jiangxi Province	450	N 186	115.7383	28.79894
Xingguo County, Jiangxi Province	190	N 187	115.0963	26.32225
Xiushui County, Jiangxi Province	660	N 188	114.1676	28.83247
Xiushui County, Jiangxi Province	400	N 189	114.7697	28.84127
Xiushui County, Jiangxi Province	50	N 190	114.6836	29.00868
Xiushui County, Jiangxi Province	445	N 191	114.8322	29.04223
Xiushui County, Jiangxi Province	260	N 192	114.8089	28.81911
Yongxin County, Jiangxi Province	342	N 193	114.0897	27.14444
Zixi County, Jiangxi Province	259	N 194	108.9635	27.36328
Danfeng County, Shaanxi Province	1100	N 195	110.4395	33.88827
Lueyang County, Shaanxi Province	822	N 196	106.071	33.4671
Lueyang County, Shaanxi Province	850	N 197	106.4453	33.45441
Pingli County, Shaanxi Province	520	N 198	109.3333	32.31667
Pingli County, Shaanxi Province	850	N 199	109.1257	32.25593
Xi'an City, Shaanxi Province	400	N 200	109.0297	34.20941
Yang County, Shaanxi Province	1400	N 201	107.5445	33.58814
An County, Sichuan Province	1280	N 202	104.2151	31.74102
Chengdu City, Sichuan Province	535	N 203	103.8076	30.68576
Chengdu City, Sichuan Province	700	N 204	103.839	30.86533
Emeishan City, Sichuan Province	1200	N 205	103.3839	29.57902
Gao County, Sichuan Province	1200-1500	N 206	104.5927	29.17474
Gao County, Sichuan Province	640	N 207	104.4087	28.35078
Gulin County, Sichuan Province	670	N 208	105.8453	28.29056
Guan County, Sichuan Province	550	N 209	103.6232	30.99605
Hanyuan County, Sichuan Province	1200	N 210	102.727	29.34815
Hongya County, Sichuan Province	1000	N 211	103.2311	29.71896
Huili City, Sichuan Province	2300	N 212	102.0497	26.58689

Jinyang County, Sichuan Province	1500	N 213	103.2491	27.69867
Kangding County, Sichuan Province	1575	N 214	102.1839	30.11518
Luding County, Sichuan Province	1770	N 215	102.1159	29.61904
Luding County, Sichuan Province	1900	N 216	102.1129	29.62207
Luding County, Sichuan Province	1800	N 217	102.1256	29.63987
Nanjiang County, Sichuan Province	80	N 218	106.753	32.2079
Pingwu County, Sichuan Province	861	N 219	104.5555	32.40968
Pingwu County, Sichuan Province	1500	N 220	104.3405	32.37629
Pingshan County, Sichuan Province	553	N 221	104.0329	28.78226
Pingshan County, Sichuan Province	350	N 222	104.3082	28.80922
Pingshan County, Sichuan Province	831	N 223	104.0329	28.78226
Pingshan County, Sichuan Province	1000-1400	N 224	103.8502	28.77266
Puge County, Sichuan Province	450	N 225	102.6132	27.3113
Qingchuan County, Sichuan Province	800	N 226	105.2385	32.57582
Tongjiang County, Sichuan Province	1350	N 227	107.5544	32.02847
Wenchuan County, Sichuan Province	1800	N 228	103.2207	31.07473
Xingwen County, Sichuan Province	800	N 229	105.3091	28.21373
Xingwen County, Sichuan Province	800	N 230	104.9402	28.24729
Xingwen County, Sichuan Province	1300	N 231	105.0305	28.24741
Xuyong County, Sichuan Province	1050	N 232	105.3656	28.15389
Xuyong County, Sichuan Province	1015	N 233	105.5189	28.27028
Xuyong County, Sichuan Province	300-500	N 234	105.2614	28.16222
Xuyong County, Sichuan Province	1500	N 235	105.6012	28.24075
Xuyong County, Sichuan Province	356	N 236	105.5392	28.31161
Xuyong County, Sichuan Province	1500	N 237	105.4647	27.80701
Xuyong County, Sichuan Province	1015	N 238	105.5189	28.27028
Ya'an City, Sichuan Province	631	N 239	103.3445	30.12335
Ya'an County, Sichuan Province	1000	N 240	103.1183	29.81741
Zizhong County, Sichuan Province	1050	N 241	105.0761	29.36669
Qimen County, Anhui Province	-	N 242	117.5	29.5
Shexian County, Anhui Province	-	N 243	118.43	29.86
Anlong County, Guizhou Province	-	N 244	105.39	25.13
Pingba County, Guizhou Province	-	N 245	106.25	26.4
Weng'an County, Guizhou Province	-	N 246	107.4	27.17
Songtao Miao Autonomous County, Guizhou Province	-	N 247	109.2	28.15
Zunyi City, Guizhou Province	-	N 248	106.9091	27.65832
Dejiang County, Guizhou Province	-	N 249	108.1199	28.26412
Guiyang City, Guizhou Province	-	N 250	106.7611	26.54349
Changshun County, Guizhou Province	-	N 251	106.4115	25.9946
Zunyi County, Guizhou Province	-	N 252	106.8299	27.53623
Qingzhen City, Guizhou Province	-	N 253	106.337	26.67972
Tongzi County, Guizhou Province	-	N 254	106.8256	28.13355
Song County, Henan Province	-	N 255	112.03	33.96

Xiayi County, Henan Province	-	N 256	116.13	34.23
Xinyang City, Henan Province	-	N 257	114.08	32.1
Lushi County, Henan Province	-	N 258	111.0479	34.0542
Zhengzhou City, Henan Province	-	N 259	113.6582	34.78038
Fang County, Hubei Province	-	N 260	110.71	32.14
Badong County, Hubei Province	-	N 261	110.34	31.04
Lichuan City, Hubei Province	-	N 262	108.93	30.29
Xuan'en County, Hubei Province	-	N 263	109.48	29.99
Jianshi County, Hubei Province	-	N 264	109.72	30.6
Zhuxi County, Hubei Province	-	N 265	109.8157	32.08939
Wuchang City, Hubei Province	-	N 266	114.316	30.55386
Hubei province	-	N 267	112	31
Hunan Province	-	N 268	113.5745	28.40766
Hunan Province	-	N 269	111.3167	26.46667
Shimen County, Hunan Province	-	N 270	111.02	29.69
Xinning County, Hunan Province	-	N 271	110.85	26.43
Baojing County, Hunan Province	-	N 272	109.65	28.7
Xinshao County, Hunan Province	-	N 273	111.45	27.32
Yongshun County, Hunan Province	-	N 274	109.85	29
Cili County, Hunan Province	-	N 275	111.13	29.42
Xinning County, Hunan Province	-	N 276	110.85	26.43
Dongkou County, Hunan Province	-	N 277	111.4819	27.23979
Jiangyong County, Hunan Province	-	N 278	111.3439	25.27354
Ruichang City, Jiangxi Province	-	N 279	115.41	29.62
Lushan City, Jiangxi Province	-	N 280	115.9892	29.67178
Xiushui County, Jiangxi Province		N 281	114.4126	29.0101
Fenyi County, Jiangxi Province	150	N 282	114.6681	27.81111
Shaanxi Province	-	N 283	107.0455	33.08157
Yang County, Shaanxi Province	-	N 284	107.5455	33.22
Lueyang County, Shaanxi Province	-	N 285	106.15	33.32
Danfeng County, Shaanxi Province	-	N 286	110.3273	33.69578
Mian County, Shaanxi Province	-	N 287	106.673	33.15355
Liuba County, Shaanxi Province	-	N 288	106.9212	33.61776
Baoji City, Shaanxi Province	-	N 289	106.9516	34.42674
Meixian County, Shaanxi Province	-	N 290	107.844	34.15745
Luding County, Sichuan Province	-	N 291	102.2346	29.91416
Xingwen County, Sichuan Province	-	N 292	105.1096	28.2701
Yibin County, Sichuan Province	-	N 293	104.37	28.83
Emeishan City, Sichuan Province	-	N 294	103.3986	29.49612
Leibo County, Sichuan Province	-	N 295	103.5717	28.26272
Leibo County, Sichuan Province	-	N296	102.8467	29.79293
