# Supplementary material for: Relationship between secondary metabolites and ecological suitability zones for Eucommia ulmoides
Source: PLoS One. 2025 Jan 30;20(1):e0317368. doi: 10.1371/journal.pone.0317368 (PMC11781706; doi:10.1371/journal.pone.0317368)
Supplement: S2 Table — (PDF) [file pone.0317368.s005.pdf]

S2 Table Variables used in the model prediction

Variable description	Abbreviated name	Unit
Annual mean temperature	Bio-1	℃
Mean diurnal range (Mean of monthly (max.temp． -min.temp．))	Bio-2	℃
Isothermality [Bio2 /(Bio5-Bio6)](×100)	Bio-3	%
Temperature seasonality (Standard deviation ×100)	Bio-4	%
Max temperature of warmest month	Bio-5	℃
Min Temperature of Coldest Month	Bio-6	℃
Mean temperature of wettest quarter	Bio-8	℃
Annual precipitation	Bio-12	mm
Coefficient of variation of precipitation seasonality	Bio-15	%
Precipitation of warmest quarte	Bio-18	mm
Topsoil Gravel Content	T_GRAVEL	%vol.
Topsoil Sand Fraction	T_SAND	% wt.
Topsoil Silt Fraction	T_SILT	% wt
Topsoil Reference Bulk Density	T_REF_BULK	kg/dm^3
Topsoil Bulk Density	T_BULK_DEN	kg/dm^3
Topsoil Organic Carbon	T_OC	% weight
Topsoil CEC (clay)	T_CEC_CLAY	cmol/kg
Topsoil CEC (soil)	T_CEC_SOIL	cmol/kg
Topsoil TEB	T_TEB	cmol/kg
Topsoil Calcium Carbonate	T_CACO3	% wt.
Topsoil Gypsum	T_CASO4	% wt.
Topsoil Sodicity (ESP)	T_ESP	%
Subsoil Gravel Content	S_GRAVEL	%vol.
Subsoil Clay Fraction	S_CLAY	% wt.
Subsoil Reference Bulk Density	S_REF_BULK	kg/dm^3
Subsoil Bulk Density	S_BULK_DEN	kg/dm^3
Subsoil Organic Carbon	S_OC	% wt.
Subsoil CEC (soil)	S_CEC_SOIL	cmol/kg
Subsoil TEB	S_TEB	cmol/kg
Subsoil Gypsum	S_CASO4	% wt.
Altitude	Alt	m
Aspect	Asp	—
Slope	Slp	°
